# Modeling Damage Complexity-Dependent Non-Homologous End-Joining Repair Pathway

**DOI:** 10.1371/journal.pone.0085816

**Published:** 2014-02-10

**Authors:** Yongfeng Li, Pamela Reynolds, Peter O'Neill, Francis A. Cucinotta

**Affiliations:** 1 Division of Space Life Sciences, Universities Space Research Association, Houston, Texas, United States of America; 2 Department of Oncology, Gray Institute for Radiation Oncology & Biology, University of Oxford, Oxford, United Kingdom; 3 University of Nevada, Las Vegas, Department of Health Physics and Diagnostics Sciences, Las Vegas, Nevada, United States of America; 4 NASA, Lyndon B. Johnson Space Center, Space Radiation Program, Houston, Texas, United States of America; Tulane University Health Sciences Center, United States of America

## Abstract

Non-homologous end joining (NHEJ) is the dominant DNA double strand break (DSB) repair pathway and involves several repair proteins such as Ku, DNA-PKcs, and XRCC4. It has been experimentally shown that the choice of NHEJ proteins is determined by the complexity of DSB. In this paper, we built a mathematical model, based on published data, to study how NHEJ depends on the damage complexity. Under an appropriate set of parameters obtained by minimization technique, we can simulate the kinetics of foci track formation in fluorescently tagged mammalian cells, Ku80-EGFP and DNA-PKcs-YFP for simple and complex DSB repair, respectively, in good agreement with the published experimental data, supporting the notion that simple DSB undergo fast repair in a Ku-dependent, DNA-PKcs-independent manner, while complex DSB repair requires additional DNA-PKcs for end processing, resulting in its slow repair, additionally resulting in slower release rate of Ku and the joining rate of complex DNA ends. Based on the numerous experimental descriptions, we investigated several models to describe the kinetics for complex DSB repair. An important prediction of our model is that the rejoining of complex DSBs is through a process of synapsis formation, similar to a second order reaction between ends, rather than first order break filling/joining. The synapsis formation (SF) model allows for diffusion of ends before the synapsis formation, which is precluded in the first order model by the rapid coupling of ends. Therefore, the SF model also predicts the higher number of chromosomal aberrations observed with high linear energy transfer (LET) radiation due to the higher proportion of complex DSBs compared to low LET radiation, and an increased probability of misrejoin following diffusion before the synapsis is formed, while the first order model does not provide a mechanism for the increased effectiveness in chromosomal aberrations observed.

## Introduction

The induction of DNA double strand break (DSB) by ionizing radiation and other agents can lead to cell death and mutation if not repaired efficiently, and are associated with genomic instability and cancer risk. One of the most important DNA repair pathways is non-homologous end-joining (NHEJ) which is utilized by the majority of DSBs, whereas replication-induced DSBs, formed at stalled replication forks, are normally repaired by homologous recombination (HR). In addition to the classical NHEJ pathway, cells may also use a Ku-independent back-up NHEJ pathway which involves poly(ADP)ribose polymerase (PARP1) and ligase III [2], [3], This back-up NHEJ pathway was verified not to play a substantial role in DSB repair in Ku70/80 proficient cells [1], [3]. There are numerous NHEJ proteins including Ku70/80, DNA-PKcs/Artemis, XRCC4/Ligase IV, XLF, etc. [4], [5]. The classical sequential model of NHEJ assumes that once induced by ionizing radiation (IR), a DNA end will first recruit Ku, and then DNA-PKcs followed by other repair proteins [6]. In contrast, the two-phase model suggested that, except Ku, the recruitment ordering of DNA-PKcs and other proteins does not matter [7].

More recently, Mari *et al* [2] and Yano *et al* [8] presented indications that a fraction of DSBs may be repaired by NHEJ in a Ku70/80-dependent DNA-PKcs-independent manner. Only few real time studies have focused at longer times mainly on the dependence of the kinase activity of DNA-PKcs on the kinetics of DSB repair [9]–[11]. Both ATM phosphorylation and autophosphorylation of DNA-PKcs were found to be essential for efficient DSB repair [12] by facilitating release of DNA-PKcs from DNA ends [9]. Recently it was demonstrated that the repair of DSBs by NHEJ is highly regulated with pathway choice and kinetics of repair dependent on the complexity of the DSBs [1]. Further, it was shown [1] that DSBs with greater chemical complexity are repaired slowly involving not only Ku70/80 and XRCC4/Ligase IV/XLF but also require DNA-PKcs. ATM inhibition only retards repair of the more chemically complex DSBs [1]. Evidence for the inefficient repair of chemically complex DSBs also came from findings where an increased number of persistent DSBs was observed [13]–[15] in cell lines deficient in either, Artemis (involved in NHEJ) or ATM (involved in DSB signaling and NHEJ). Recent biochemical studies provided further insights into complex DSB repair, confirming the inefficient processing of chemically complex DSBs [16], [17].

On the other hand, from the kinetics of rejoining of DSB determined by physical methods such as PFGE [18], [19], it has been observed that the DNA repair process exhibits at least a biphasic profile, indicating that a proportion of DSBs are rejoined through fast kinetics with the remainder by slow kinetics. Indeed the rate of loss of fluorescently-tagged XRCC4-GFP from laser-induced simple DSB [1] is comparable with the rate of repair of the majority of γ-radiation-induced DSBs, determined by PFGE [19]. The mechanism behind this biphasic kinetics, however, is still under debate. Together with the above mentioned complexity of DNA damage hypothesis for the biphasic kinetics other hypotheses have been put forward. The first one is related to the length of IR induced DNA fragments, suggesting that short fragments have lower efficiency of recruiting repair proteins, such as Ku, and hence lower repair rate than long fragments [20]. A stochastic model was proposed to support this argument [21]. The second mechanism excludes the types of DNA damage and emphasizes the roles of chromatin, showing that the DNA damage undergoes slower repair in the heterochromatin due to its compact structure with less accessibility to the repair proteins than that in the euchromatin which has more relaxed structure [22]. However, several studies have shown that the recruitment of proteins to sites of DNA damage is essentially independent of the chromatin compaction but may reflect changes in the diffusion coefficients related to the packing density of the chromatin [23]–[25]. Direct determination of the rejoining rates of DSB was shown to be similar in both euchromatin and heterochromatin [26].

Several mathematical models have been proposed to study a DNA repair complex and the NHEJ repair pathway in terms of biochemical kinetics [27]–[35]. These models are based on the rate of repair of DSBs post irradiation, as measured by PFGE (generally in the dose range of 40–100 Gy), where the rate determining step essentially defines the rate of DSB rejoining, generally ligation of the majority of DSB for low LET radiation. Li and Cucinotta [28] used mathematical analysis to study the importance of the sequence of recruitment of several repair proteins in NHEJ. More recently, Taleei and Nikjoo [34], [35] have modeled NHEJ repair kinetics from biochemical data in repair proficient and deficient cells following high IR doses (10–80 Gy). Additionally, they have modeled recruitment kinetics of Ku80 and DNA-PKcs from real time kinetic data [2], [8] albeit over a short time. The study by Reynolds *et al* [1] was able to comment in more depth from real time kinetics on the kinetics of the individual repair steps of DSB by NHEJ through determination of the rates of recruitment and loss of the various proteins. Here we have used these experimental data on real time protein dynamics for some of the individual steps during NHEJ over long times post irradiation to develop a mathematical model of NHEJ.

In this study, the aim was to determine whether the complexity of DNA damage affects the dynamics and proteins involved in DNA repair through development of refined mathematical models of NHEJ by considering the simple and complex DSBs as different substrates requiring different protein components of the NHEJ pathway. As proposed in [1], [2], [8] and indirectly in [13]–[15], simple DSB recruits Ku and XRCC4/ligase IV for direct repair, while complex DSB first recruits Ku and DNA-PKcs for end processing, then XRCC4/ligase IV for repair. This approach can be modeled in terms of a sequence of chemical reactions and governed by a system of ordinary differential equations by applying law of mass action. We used the gradient method to search for the appropriate values of the involving parameters such that the numerical simulation of the model has a good fit to the experimental data related to the kinetics of formation of Ku80-EGFP and DNA-PKcs-YFP at sites of DNA damage induced by ultrasoft X-rays (USX) or multi-photon IR induced laser light described in [1]. Whereas USX produce mainly simple DSB, the high powers generally used with NIR lasers result in a high density of damage, as discussed in reference [36], in the range of high LET charge particles as also suggested by Splinter *et al* [37] and previously proposed from laser studies [38], [39]. By fixing all the parameters except those related to ATM, the model also fits the data when an ATM inhibitor is used to retard the phosphorylation and hence release of DNA-PKcs. Therefore our model supports the mechanism proposed in [1] that the repair of simple and complex DSBs can be distinguished by the absence and presence of DNA-PKcs and hence the inhibition of ATM lowers the phosphorylation efficiency of DNA-PKcs, affecting the rate of repair of complex DSBs, but not of simple DSBs. It has previously been proposed [13] that ATM plays a facilitating role in DSB but is also required more directly for repair of a sub-set of DSB.

In the model, we proposed that DSB repair can be modeled in different ways, through either break filling (BF) which leads to a first order model, or synapsis formation (SF) [40]. By comparison, we found that a SF model provides an improved fit to experimental data compared to the BF model, suggesting that DSB repair is through synapsis formation more likely than break filling for complex DSB, whereas the repair of simple DSB has a higher BF rate than that for complex DSB. These differences in the use of BF and SF provide an alternate explanation of why complex DSBs are repaired more slowly than simple DSB, in addition to the extra step of end processing and ATM-dependent phosphorylation and release of DNA-PKcs [1], [9], [11], [12].

## Methods

### Summary of Experimental Conditions

The biological experiments have been reported in [1]. In this section, we briefly introduced the experimental design and observation so that a comparison of the model with data is easily accessible. We refer the readers to [1], [38], [39] for more details on the experimental data and the laser microbeam system characterization and set-up.

In the experiments, DSBs were induced in fluorescently tagged mammalian cells (Ku80-EGFP tagged XR-V15B cells and DNA-PKcs-YFP tagged V3 cells) by using ultra-soft X-rays or multi-photon near infrared (NIR) laser microbeam radiation. Ku80-EGFP cells were cultured in minimum essential medium (MEM) supplemented with 2 mM L-glutamine and DNA-PKcs-YFP cells were cultured in αMEM containing glutamax. All cell culture medium was supplemented with 10% FCS and 100 units/ml penicillin and 100 µg/ml streptomycin in T75 flasks. Cells were plated at 2.0×10^5^ cells/dish in 30 mm diameter glass walled, number 1 glass cover-slip bottom dishes containing 3 ml of medium and incubated for 24 hours at 37°C and 5% CO_2_ humidified air. The expression levels of Ku80-EGFP and DNA-PKcs-YFP have been shown to be similar to that of the respective protein in the wild-type cells [1], [41], [42].

Cells were incubated with 10 µg/ml Hoechst dye for 10 minutes prior to irradiation at 37°C and maintained at 37°C throughout the irradiation. The laser was set to be a wavelength of 730 nm and a nominal power of 10 mW. Time zero was recorded immediately following irradiation (<10 seconds) and images were collected at the stated time points using BioRad Radiance 2000 confocal microscopy coupled to a Nikon TE2000 microscope. To study the effect of ATM, 10 µM ATM inhibitor was added 45 minutes prior to damage induction. The inhibitor concentration was chosen according to either IC_50_ or EC_50_ and recommended by the manufacture.

The experimental study has concluded that the choice of the NHEJ repair pathway for the different types of DSBs is independent of the phase of the cell cycle. DSBs recruit one Ku80 molecule per end so that for the experiment to observe the damage requires resolution of individual foci- which are more adequately described as “foci tracks”. Experimental conditions are such that foci tracks are formed at doses needed to ensure the concentration of Ku protein in the track is greater than the concentration of the background due to the abundance of Ku in cells.

### Model Development

Ionizing radiation produces DNA damage in the form of simple or complex DNA double strand breaks (DSBs), the majority of which are repaired through non-homologous end joining (NHEJ) pathway. Once DSBs are generated, Ku will be recruited to the DSBs and bound with the DNA free ends. It is proposed in [1], [2], [8] that the complexity of the DSB determines the choice of NHEJ repair pathway and necessary biochemical steps which involved different NHEJ components. Precisely, simple DSBs recruit repair proteins involved in ligation such as XRCC4 and Ligase IV once Ku has been recruited so that DSBs are rejoined after Ku release followed by the subsequent release of the repair proteins involved in ligation. In contrast, complex DSBs additionally require DNA-PKcs for additional processing of dirty end (removal of lesions close to the ends and overhangs) involving the recruitment of additional repair proteins. Afterwards, the release of DNA-PKcs is through its autophosphorylation and enhanced by its phosphorylation by ATM, which has been confirmed by experiments showing that ATM inhibition leads to the retention of DNA-PKcs on the DSBs as previously shown [11] and reduces the repair efficiency of complex DSBs [1], [13].

According to the working model proposed in [1] and the earlier indications of Mari *et al* [2] and Yano *et al* [8], we have built mathematical models to study the DSB repair by complexity-dependent NHEJ pathways. We considered the classical NHEJ repair pathway, in which Ku, DNA-PKcs, Artemis, XRCC4, Ligase IV and XLF are mainly involved. We treat DNA-PKcs/Artemis as a single species in the model of chemical reactions [43]. XRCC4 usually functions with Ligase IV and also forms a tight complex and stabilize each other [44]. XLF is an XRCC4-like factor. The role of XLF is not quite clear, but experimental study suggested that XLF can stabilize XRCC4/Ligase IV at DSB to promote the DNA ligation [45]. We have studied NHEJ by mathematical modeling the reactions of all these individual DSB agents and concluded that the classical sequential model and two-phase model share similarity, in other word, the recruitment order of DSB agents (except Ku) does not matter [21]. On the other hand, our goal is to study the repair of simple and complex damage in which Ku and DNA-PKcs play key roles, therefore, we considered only three agents Ku, DNA-PKcs (assumed to be bound with Artemis) and XRCC4 (assumed to be bound with Ligase IV and XLF and denoted by XL).

**Figure 1:**
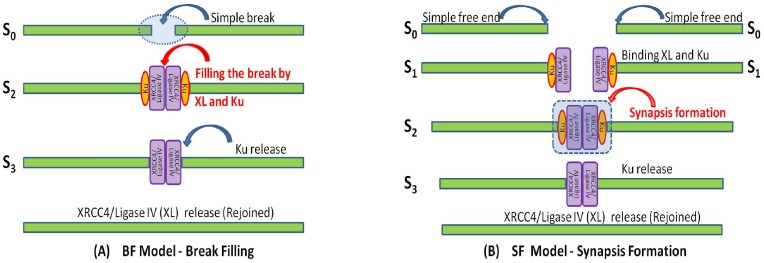
Modeling the repair of simple DNA double strand breaks. DSBs repair can be modeled in terms of either (A) Break filling in which the break between two nearby DNA ends remains and is filled by NHEJ proteins for ligation and repair. S_0_ represents the simple break, S_2_ the break filled by Ku and XRCC4/Ligase IV (XL) and S_3_ the filled break with Ku released. (B) Synapsis formation in which the break may become a larger gap due to diffusion and requires the extra roles of NHEJ protein to tether and rejoin the DNA ends, and S_0_ represent the simple free DNA end, S_1_ simple DNA end bound with Ku and XL, S_2_ the synapsis fromed by two simple DNA ends and S_3_ the synapsis with Ku released.

Although the mechanism of NHEJ is straightforward, the details can be modeled in different ways, as shown in Figure 1, where two examples for DSB repair are given, namely BF and SF models. For a single DNA fragment, one DSB results in two pieces of shorter fragments. If the two resulting fragments remain where they are generated without significant diffusion, then the break remains and is filled by the recruited NHEJ proteins for repair (see Figure 1-A). In this paper, we call the DNA repair model through break filling (BF) a BF model, in which the number of breaks is counted and reduced by one after one break is repaired. If, however, the resulting fragments diffuse, the two nearby DNA free ends may separate and need to be rejoined under the help of binding NHEJ proteins. Before DSB repair, two DNA ends bound with NHEJ proteins must meet to form a synapsis. The synapsis formation (SF) rate relies heavily on the probability that two DNA ends meet before rejoining, and hence the concentration of DNA ends, instead of DNA breaks. To distinguish the DNA repair through the break filling, the DNA repair model through synapsis formation is called a SF model (see Figure 1-B).

Figure 1 shows the BF model and the SF model for the simple DSB repair. In both BF and SF models, S_2_ represents a high order complex consisting of two DNA ends held together by Ku and XRCC4/Ligase IV (XL), and S_3_ the same complex but with Ku having been released. However, such a complex S_2_ is generated in different ways for the two models. In the BF model, S_2_ arises directly from the filling of a simple DNA break and S_0_ by Ku and XL (see Figure 1-A). In contrast, in the SF model, two free DNA ends are present so that S_0_ results from a break initially binding with Ku and XL to form DNA complex S_1_, then two resulting complexes meet to form a synapsis S_2_ (see Figure 1-B). For convenience in writing the chemical reaction system and compare the models, here we use the notation for S_0_ for the two different types of breaks, simple DNA break in the BF model and simple free DNA end in the SF model. As seen later, such subtle differences in formulations lead to different models whose output fit the experimental data with different levels of accuracy.

**Figure 2:**
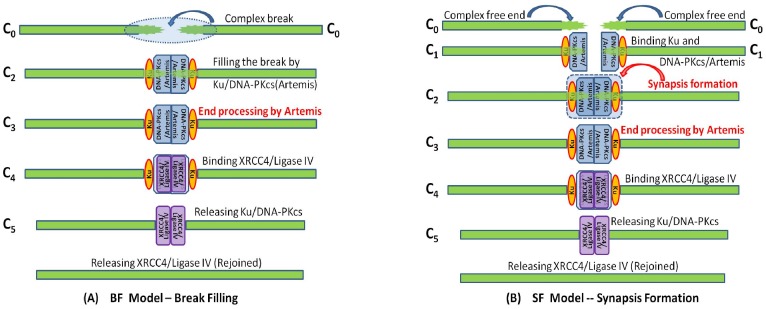
Modeling the repair of complex DNA double strand breaks. DSBs repair can be modeled in terms of either: (A) break filling in which C_0_ represent the complex break, C_2_ the break filled by Ku and DNA-PKcs/Artemis and C_3_ the filled break after end processing by Artemis, C_4_ the filled break bound with XL, and C_5_ the filled break with Ku and DNA-PKcs released; or (B) synapsis formation in which C_0_ represent the complex free DNA end, C_1_ complex DNA end bound with Ku and DNA-PKcs/Artemis, C_2_ the synapsis fromed by two complex DNA ends, C_3_ the synapsis after end processing by Atemis, C_4_ the synapsis with XL recruited, and C_5_ the synapsis with Ku and DNA-PKcs released.

The repair of complex DSBs can be modeled similarly (Figure 2), but the break filling and synapsis formation are conducted by binding Ku and DNA-PKcs/Artemis, instead of only Ku and XL for simple DSB. Moreover, as shown in Figure 2, complex DSB repair requires at least two more extra steps: First, DNA-PKcs tethers the DNA free ends to prevent their diffusion, and Artemis (activated by DNA-PKcs phosphorylation) helps clean up the DNA ends so that a pair of DNA ends bound with DNA-PKcs/Artemis form synapsis before the ends rejoining [46]. Second, DNA-PKcs must be released after end processing so that the recruited repair proteins XL have access to the DNA ends for rejoining.

In the following, by sDSB and cDSB we denote the DNA free ends resulting from simple and complex DSBs, respectively. In summary, we list all the new notations, introduced above, in Table 1 to represent the complexes involved in the NHEJ repair pathway of simple and complex DSBs.

**Table 1:** Notation for the complexes involved in the NHEJ repair of simple and complex DSBs.

Symbol	XL	S_0_	S_1_	S_2_	S_3_	
Complex	XRCC4/Lig IV	sDSB	sDSB/Ku/XL	S-Synpasis	S_2_ w/o Ku	
	**C_0_**	**C_1_**	**C_2_**	**C_3_**	**C_4_**	**C_5_**
Complex	cDSB					C_4_ w/o DNAPK

Moreover, we assume that

(A1) Ku is released simultaneously with DNA-PKcs in the complex DSB repair;

(A2) All the NHEJ proteins are abundant and remain constant concentrations.

It is known that DNA-PKcs binds with Ku to form DNA-PK and DNA-PKcs is released due to its conformational change under autophosphorylation. So it is reasonable to assume Ku is released together with DNA-PKcs in the complex DSB repair. In addition, it has been reported that Ku is abundant (over one-half million copies in one cell) [47] and DNA-PKcs is also moderately abundant [43]. In the experiments [1], we standardized at a dose of 27 Gy which produces about 400–500 DSBs. These yields of DSB are very low when compared to the copy number of Ku molecules per cell of several hundred thousand as quoted. Other NHEJ proteins may not be so abundant as Ku and DNA-PKcs. But because we are to compare with data of Ku and DNA-PKcs instead of XRCC4, the kinetics does not change much regardless of constant or kinetic concentration. Therefore we assume that all the repair proteins including Ku, XRCC4/Ligase IV (XL) and DNA-PKcs are abundant relative to the concentration of DSB and that their concentrations remain constant, then associated biochemical reactions are as given as follows
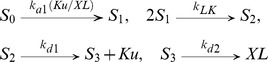
(1)

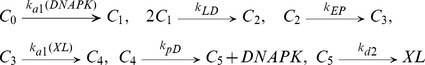
(2)where we assume the DSB transits to a higher order complex by binding Ku, DNA-PKcs, XL at the same rate k_a1_ to the DNA free end as all the NHEJ proteins are recruited to DSB on the same time scale within a minute [1], [2], [11]; k_d1_ and k_d2_ are the release rates of Ku and XL from DSB, k_EP_ the end processing rate of complex DNA end by DNA-PKcs, k_LK_ and k_LD_ the SF rates of simple and complex DSBs and k_pD_ the release rate of DNAPK (complex of DNA-PKcs and Ku) through the autophosphorylation of DNA-PKcs and its phosphorylation by ATM. It was proposed that Ku is released when DNA-PKcs is removed due to phosphorylation [1], [28], while recent studies suggested that Ku is released through DSB-induced ubiquitination [48] or by Mre11 nuclease activity [41]. Here, we simply assume that Ku is released independent of DNA-PKcs in the repair of simple DSB, but together with DNA-PKcs through its autophosphorylation in the repair of complex DSB. Once the complex DNA ends are processed, the recruitment of XRCC4/Ligase IV occurs to a DSB that by this time will be similar to a simple DSB end. Thus we assume that both simple and complex DSB repair show the same recruitment and release rates of XL once the complex ends have been ‘cleaned-up’.

Note that the boxed reactions in systems (1–2) indicate the synapsis formation of simple and complex DSBs. The removal of these two reactions leads to the BF model by identifying S_1_ with S_2_, and C_1_ with C_2_. In the BF model, all the species S and C represent the DNA DSB, instead of DNA DSB ends. Indeed, both the BF and the SF models lead to the same long term dynamics in the sense that all the DSBs are repaired (rejoined) eventually. As seen later, however, the subtle biological difference between break filling and synapsis formation shows variation in the early time kinetics, when the comparison with experimental data is mainly conducted. Precisely, the SF model predicts the experimental results [1] in a satisfactory way whereas the BF model does not. Thus, we will only consider the SF model in the subsequent considerations. The details for both models can be found in the Supplementary Material File S1.

After having the biochemical based model (1–2), we need to build a mathematical model to simulate the kinetics of this reaction system numerically to reveal its qualitative features. For this purpose, we had to apply some laws of chemical reaction, such as mass action, that provides a connection between the kinetic changes, products, and concentrations of the associated reactants. Again, we also let S_i_ and C_i_ denote the concentrations of the species they represent. In order to have a compact form of the model in a systematic way, we set ***X*** = (**X**_S_,**X**_C_) where **X**_s_ = (S_0_,S_1_,S_2_,S_3_) and **X**_C_ = (C_0_,C_1_,C_2_,C_3,_C_4_,C_5_) are the species involving in the repair of simple and complex DSBs, respectively. Then the levels of Ku80-EGFP and DNA-PKcs-YFP recruited to DSB can be defined by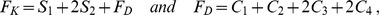 respectively. To mimic the kinetic change of the levels of various repair proteins at the DSB sites to solve the above system, we also need data about the distribution or production kinetics of simple and complex DSBs. It is known that DSBs are induced not only during the period when radiation is applied, but also in the post-radiation period. For example, DSBs can be generated during the processing of clustered DNA damage in the mammalian cells post-irradiation [19]. In this model, we have considered only the direct induction of DSB by radiation and not included any post-radiation induced DSB, as many will not utilize the NHEJ pathway for processing. Other than the initial distribution of simple and complex DSBs, the dynamical production of DSBs induced by radiation is considered by taking into account the irradiation time T_R_. In other words, assuming the production rates of simple and complex DSBs are b_S_ and b_C_ and the dose-rate is sufficiently high with respect to the repair time for complex DSB, we have two additional reactions
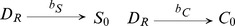
Where D_R_ is the dose rate. Note that D_R_ can be given in terms of Heaviside function H(t), that is H(t) = 0 as 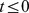 and H(t) = 1 when t>0. For instance, we may set D_R_(t) = b_S_ H(T_R_−t) for the acute dose or D_R_(t) = b_S_ [H(T_2i+1_−t)−H(T_2i−t_)] for the fractionated doses, where 0 = T_0_<T_1_<T…<T_2M−1_ = T_R_. Therefore, the reaction rate equations of NHEJ repair of DSB can be written in terms of a system of differential equations
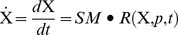
(3)where **SM** is the associated stoichiometric matrix, and **R**(**X**,**p**,t) the reaction fluxes vector and **p** is the parameter vector. See Supplementary Material File S1 for details. The recruitment of NHEJ proteins within the radiation time is usually ignored when using laser for damage induction in [1] because of its short duration of about 30 s. But it should be counted when long duration radiation is applied, for example USX-rays [1] which may take up 15 minutes to achieve a sufficiently high dose. Therefore the model can be applied to a broader study of the effect induced by radiation of any type and cover the recruitment kinetics of repair proteins during the irradiation period.

### Data Fitting

The biological experiments show the kinetics of recruitment and loss of Ku80-EGFP and DNA-PKcs-YFP, based on their fluorescence intensity, to DNA DSB formed in ‘stripes’ by laser or USX irradiation [1]. For data comparison, in the model, we considered an ideal case of deterministic model according to the following assumptions

(A3) there are always 1–2 Ku molecules recruited at the DSBs and the fluorescence intensity is proportional to the level of Ku at the DNA damage stripes (see methods in reference [1]).;

(A4) the fluorescence intensity is proportional to the amount of DSBs.

(A5) all the DSBs are assumed to undergo repair.

We have assumed that the fluorescence intensity is proportional to and hence comparable with the concentration of Ku80-EGFP and DNA-PKcs-YFP at the DSB in the radiation stripes. Assuming that the relative fluorescence intensity {K_i_} (fluorescence intensity normalized to the maximum intensity) of Ku80-EGFP were observed at N_K_ time points {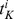} and the fluorescence intensity {D_j_} of DNA-PKcs-YFP at DSB were observed at N_D_ time points {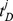}, then we have the following two data sets 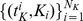 and 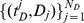. Since the experimental data in [1] was collected immediately after the cell had been irradiated with initial time set to zero, all the data time points 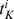 and 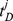 are the actual time points shifted by the radiation time T_R_ (actual experimental time points plus the irradiation time). To compare with the experimental data in [1], we set the relative fluorescence intensity of the Ku80-EGFP and DNA-PKcs-YFP at DSB on time to be
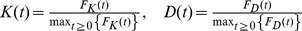
(4)Under the prescribed set of parameters q = (b_s_, b_c_, k_a1_, k_LK_, k_d1_, k_d2_, k_EP_, k_LD_) and k_pD_, we define the error function as
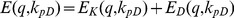
(5)where
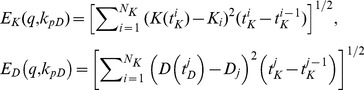
(6)are given in terms of *L^2^* norm, and 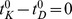 When the ATM inhibitor was included in the model and using the experimental data in [1], the dissociation rate, 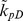 of DNA-PKcs from the DSB will be modified due to the lower phosphorylation rate *k_pD_* of DNA-PKcs from the DSB. If the associated data sets with ATM inhibition are given by 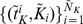 and 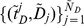, then by fixing all the parameters but *k_pD_*, the resulting error function with ATM inhibition becomes
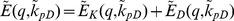
(7)and similarly we have
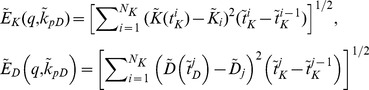
(8)And 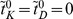. Let 
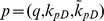
 then we have the total error function
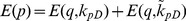
(9)To obtain the best fits to experimental data shown in [1], we have to minimize the error function over parameter *p*. By appropriate initial estimate of the parameters, we apply gradient methods (see the Supplementary Material File S1 for details), together with solving equation (3) and its adjoined system to determine the parameter p*. However, since the convexity of error function *E(p)* is not guaranteed, p* determined in this way may not be the optimal choice that minimizes the error function. Alternatively, the error function can be defined using different metrics to measure the error between the numerical solution and the experimental data. In several simulations, a least square fit is used for point-by-point comparison. In contrast, use of *L^2^* norm leads to a better fitting of the overall kinetic profile.

## Results

The following numerical simulation is undertaken with the appropriate choice of the parameters by the gradient method. Because the error function may not be convex under the constraints (3), the results from gradient iteration search may not lead to the global minimizer of the error function. However, the resulting numerical simulations do show good agreement with the experimental data in [1]. In the experiments, the DSBs were induced by the NIR laser beam in [1] that usually lasts about half minute, so we set the radiation time T_R_ = 0.5 minute in the following numerical simulations. In addition, as we have assumed that all the state variables (S_i_ and C_i_) are dimensionless number concentrations, all the parameters have units of minute^−1^.

### Synapsis Formation Model

In this section, we provide the numerical simulation of the SF model. In the classical sequential model, end processing is assumed to occur after the end rejoining (synapsis formation) [1], as proposed in the case of DNA end rejoining occurs before the dirty ends had been processed [7]. We therefore studied numerically the kinetics for both cases by switching the order between the end processing and ends rejoining to see how this influences the model outcomes.

### Model 1. Synapsis Formation before End Processing

**Figure 3:**
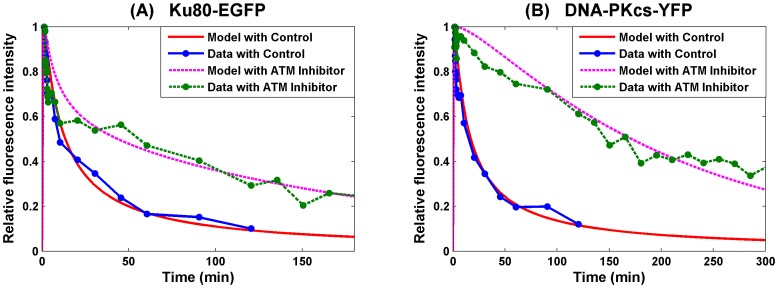
SF Model - Formation and loss of Ku80-EGFP and DNA-PKcs-YFP at the DSB in the radiation stripes with ends processing following synapsis formation. Panel (A) is for Ku80-EGFP and panel (B) for DNA-PKcs-YFP. The lines with dots are experimental data from [1], and those without dots are numerical simulation of the model. The solid lines are represent calculated normalized fluorescence intensity of the respective fluorescently tagged proteins with control, and the dashed lines are with ATM inhibitor present.

The activity of Artemis plays a key role of DNA ends processing, and the activation of Artemis requires the kinase activity of DNA-PKcs [42]. It is believed that DNA-PKcs becomes active after *in trans* autophosphorylation after the synapsis formation, and thus synapsis formation in this simulation is assumed to occur prior to end processing [1], [6], [15]. In this case, under the following set of parameters (10), we have the numerical simulation of the recruitment and loss of Ku80-EGFP and DNA-PKcs-YFP, based on their fluorescence intensity, to DNA DSB from [1] given in Figure 3. This fit for synapsis prior to end processing shows good agreement of numerical simulation with the experimental data and the error E(p) = 2.4822.
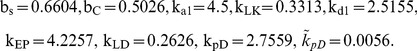
(10)


### Model 2. Synapsis Formation after End Processing

Although it has been proposed that end processing follows synapsis formation in many works [1], [6], [15], the possibility of switching the order of these two processes cannot be excluded. It was revealed that Artemis is a link between ATM activity and NHEJ [49]. Artemis is a substrate of not only DNA-PKcs [42], but also ATM. Therefore, it is reasonable to assume that DNA ends can be processed by Artemis, activated by ATM, before the synapsis is formed.

**Figure 4:**
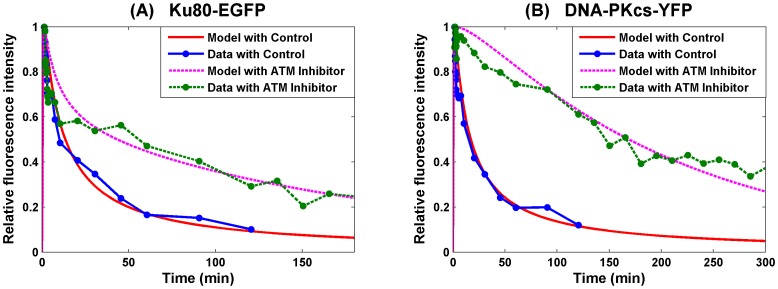
SF Model - Formation and loss of Ku80-EGFP and DNA-PKcs-YFP at the DSB in the radiation stripes with ends processing preceding synapsis formation. Panel (A) is for Ku80-EGFP and panel (B) for DNA-PKcs-YFP. The lines with dots are experimental data from [1], and those without dots are numerical simulation of the model. The solid lines are represent calculated normalized fluorescence intensity of the respective fluorescently tagged proteins with control, and the dashed lines are with ATM inhibitor present.

In this section, we consider the case that the ends processing precedes synapsis formation. Surprisingly, the gradient iteration results in very similar parameter set. And using the same set of parameter of Model 1, we have the numerical simulation to experimental data from in [1] given in Figure 4. This simulation shows a similar goodness of fit as that of Model 1 with a similar fitting error *E(p)* = 2.4855.

### Model 3. Combined Synapsis Formation and Ends Processing

**Figure 5:**
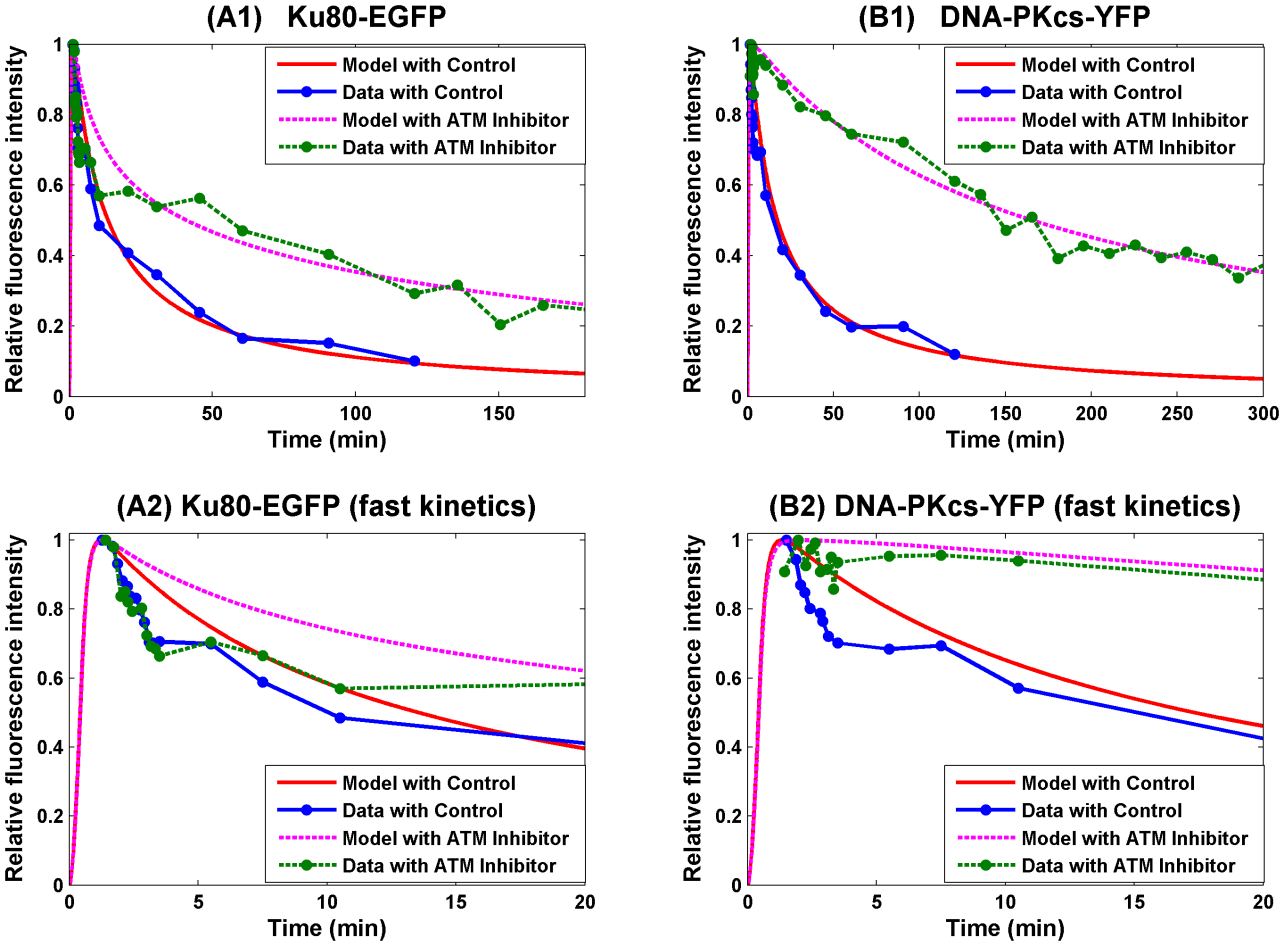
SF Model - Formation and loss of Ku80-EGFP and DNA-PKcs-YFP at the DSB in the radiation stripes with merged synapsis formation and ends processing. Panel (A) is for Ku80-EGFP and panel (B) for DNA-PKcs-YFP. The first row is for the overall kinetics and the second row is for the fast kinetics. The lines with dots are experimental data from [1], and those without dots are numerical simulation of the model. The solid lines are represent calculated normalized fluorescence intensity of the respective fluorescently tagged proteins with control, and the dashed lines are with ATM inhibitor applied.

From comparison between Model 1 and Model 2, it is suggested that the ordering of synapsis formation and end processing is not important from the viewpoint of data fitting, probably indicating that synapsis formation and end processing occur simultaneously so are not distinguishable in the model. Collectively, these two reaction steps have been combined so that,
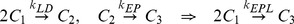
(11)The end processing is incorporated into the synapsis term with a new rate k_EP_. Furthermore, we allow a change in k_EPL_ when ATM inhibitor is included. Under the parameter set
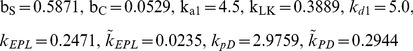
(12)this new model gives a better fit (*E(p)* = 2.1607, about 13% improvement) as shown in Figure 5.

The numerical study suggested that the combined rate of synapsis formation and ends processing is also dependent on the ATM activity. This may be due to the activity of Artemis, another important NHEJ protein. Artemis is usually attached to DNA-PKcs to form a complex [43] and is responsible for the ends processing. DNA-PKcs kinase activity is needed to activate Artemis and maintain it at the DNA damage site [42]. Moreover, Artemis is also one of the major substrates of ATM and its ATM-dependent activity is required for the ends processing that leads normally to the rejoining with slow kinetics [49]. Hence, our model (by treating DNA-PKcs and Artemis as a single complex) is in support of the extra role which ATM plays in the NHEJ through Artemis. This was not discussed in [1] in the absence of experimental evidence for real time dynamics of Artemis at DSB sites.

### Sensitivity of Parameters

**Figure 6:**
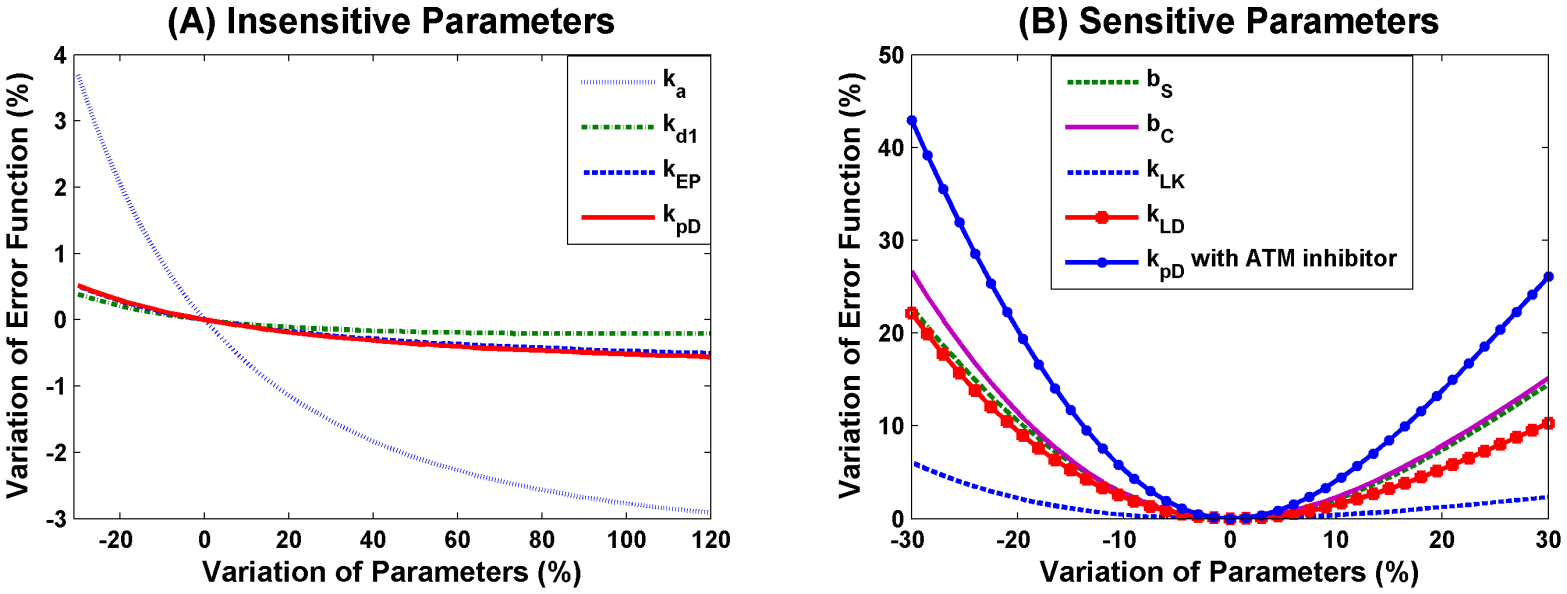
Sensitivity analysis of parameters in the SF Model 1. Let p* be the “optimal” parameter set given in scheme (10) obtained by gradient method. The x-axis presents the variation 
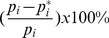
 of the parameter 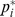 under study, and the y-axis is the resulting variation 
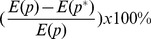
 of the data fitting error when 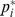 is varied but all others are fixed at the chosen values in scheme (10). Panel (A) shows four parameters whose variation by more than 30% leads to error variation by below 5%, suggesting that the model is robust to these parameters. Panel (B) includes five parameters whose variation by 30% leads to error variation by over 5%.

As observed in the above numerical simulation, the kinetics of formation and loss of Ku80-EGFP and DNA-PKcs-YFP at DSB depends on all the parameters except the release rate k_D2_ of XRCC4/Ligase IV, which is important as this is the final stage in the ligation of DSB repair. Only four parameters were considered in the double exponential model used in [1] as the best fit to the data based on first and/or second order reaction kinetics. The second exponential of this fit is less reliable as <2 half-lives were followed in the experiments due to potential photobleaching of the fluorophores for longer timescales [1]. As the presently developed model includes nine parameters to fit the experimental data in [1], it is essential to understand which parameters are crucial for the model to fit the data. For this purpose, a sensitivity analysis was undertaken as follows. Let p* be the parameter set (10) obtained by gradient method by which the SF model 1 shows good agreement with the experimental data with error equal to E(p*). If the value of one of the parameters is changed from p_i_* to *p_i_* the error will change correspondingly to become *E(p)*. In other words, variation 
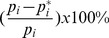
 of parameter *p_i_* leads to variation 
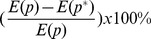
 of the overall data fitting error, which is illustrated in Figure 6 and reflects how sensitive the model is to these parameters based on the goodness of fit to the experimental data.

From Figure 6(A), we observed that around the chosen parameter set (10), the model is very robust to the recruitment rate k_a_ of NHEJ proteins, the phosphorylation rate k_pD_ of DNA-PKcs for its release and end processing rate k_Ep_ for complex DSB and the release rate k_d1_ of Ku from simple DSB. Moreover, Figure 6(A) also shows that increasing the values of these four parameters results in slightly improved fitting. Since it is not realistic to have extremely large reaction rates, we chose the values for these parameters within a reasonable range so that the model can better fit the peak time when the maximum fluorescence intensity (peak time relies heavily on k_a_, data not shown) is reached for recruitment of Ku80-EGFP and DNA-PKcs-YFP to DSB, determined in [1]. If however the rate of a specific reaction is allowed to be extremely large, then this reaction proceeds very quickly and as a consequence may not be distinguished from the other reactions. In scheme (11), as an example, a system consisting of the first two reactions can be approximated by a single reaction with k_EPL_ = k_LD_ as k_EP_ becomes large (a general discussion is provided in Supplementary Material File S1). From the modeling viewpoint, we can omit any reaction from the system if it is dispensable or merge it with other reactions if the rate is indistinguishable from that of other reactions. Consequently, the above sensitivity analysis of parameter k_EP_ provides additional evidence that it is reasonable to combine the synapsis formation and end processing into one step.

In contrast, the parameters in Figure 6(B) show larger error variations ≥30–40%. As shown in Figure 6-B, the model is most sensitive to the phosphorylation rate 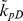 of DNA-PKcs when the ATM inhibitor is included. Note that 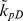
<<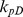, thus the sensitivity of Model 1 to the value of 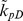 demonstrated theoretically that the experimental conclusion about the inhibitory role of ATM inhibitor in the repair of complex DSB is reasonable, particularly as stated earlier, the experimental limitations at longer times in the kinetic analysis [1]. In contrast, Model 1 is less sensitive to the induction rates (b_S_, b_C_) of simple and complex DSBs by radiation (error function shows less than 1 fold of variation when b_S_ or b_C_ undergo 30% change). The ratio b_S_∶b_C_ predicts the initial distribution of simple and complex DSBs induced by the radiation. The ratio is similar to that obtained by the double exponential kinetic fits based on the change of fluorescence intensity for loss of DNA-PKcs-YFP from DSB [1]. The sensitivity of complex SF rate k_LD_ is similar to the parameters discussed above, whilst the simple SF rate k_LK_ is the least sensitive shown in Figure 6(B). The result k_LD_<k_LK_ implies that the synapsis is formed more slowly in the repair of complex DSB than in the repair of simple DSB, additionally supporting the fact that the repair efficiency of complex DSBs is lower than that of simple DSBs. More importantly, Figure 6(B) shows that with other parameters (exhibited in Figure 6(A)) fixed, the error function reaches a local minimum value at the chosen parameters given in (12).

The sensitivity analysis of the parameters in the SF Model 3, in which synapsis formation and ends processing are merged, can be found in the Supplementary Material, see Figure S1 in File S2. In addition, to further validate the model, we have compared the numerical simulation of our model with the kinetic data of DNA-PKcs foci induced by iron ions and carbon ions [50]. Please see Supplementary Material File S2 for more details.

### Break Filling Model

**Figure 7:**
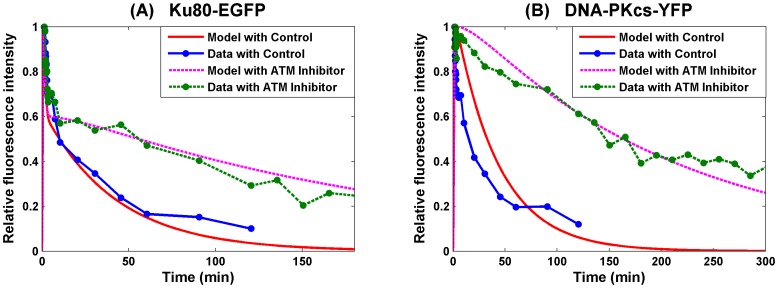
BF Model – Break Filling. Panel (A) is for Ku80-EGFP and panel (B) for DNA-PKcs-YFP. The lines with dots are experimental data from [1], and those without dots are numerical simulation of the model. The solid lines represent calculated normalized fluorescence intensity of the respective fluorescently tagged proteins with control, and the dashed lines are with ATM inhibitor present.

In this section, we assess if the BF model fits the data, and whether or not DSB repair by a break filling process also occurs. As a more a detailed model with more parameters will fit the data better, we therefore refine the first order model previously developed by separating the rate of recruitment of all NHEJ proteins. k_a1_, k_a2_, and k_a3_ represent the recruitment rates of Ku, DNA-PKcs and XL, respectively. In addition, we also assume that DNA end processing depends on the ATM activity to introduce an additional parameter 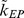 used in the first order model. The best parameter set obtained by gradient method is given below
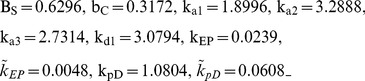
(13)Using this set of parameters, the numerical simulation of the first order model (see Appendix for details of modeling) is given in Figure 7, which shows that the model does not fit the data very well especially for DNA-PKcs in the control. The error is E(p) = 3.8994, which represents a poor fit compared with those of the second order models.

Moreover, the BF model fits the data for Ku very well within t≤60 min, in which exponential decay is reflected. This is because the BF model leads to a linear ODE system, the solution of which is given in terms of exponential functions (or multiplied by a polynomial). But beyond this time range, the model continues to exhibit exponential decay that is faster compared with that seen with the experimental data in [1]. Therefore, the BF model does not capture the long term (t>60 min) kinetics of the loss of fluorescence intensity of DNA-PKcs-YFP during DSB repair as well as the second order models do. Note that the essential difference between the BF and the SF models lies on the absence and presence of the synapsis formation. Consequently such comparison suggests that the repair of simple and complex DSBs by NHEJ is more likely second order processes of synapsis formation, rather than a first order model of merely DNA break filling.

## Discussion

It has been observed frequently in biological experiments that DNA double strand breaks (DSB) are rejoined in a biphasic manner, with fast kinetics and slow kinetics based on loss of DSB using physical methods generally based on DNA size changes [13]–[15] or through real-time imaging of fluorescently tagged repair proteins [36]. Recent findings [1] together with work [2], [8] have provided important evidence that this biphasic profile is due to the complexity of DSBs, indicating that simple DSB can be repaired quickly without DNA-PKcs but complex DSB is repaired slowly requiring DNA-PKcs. In this work, we have proposed some mathematical models to assess how the complexity of DNA damage affects the efficiency of DNA repair leading to the biphasic repair kinetics. The models are given in terms of a system of ordinary differential equations for a sequence of biochemical reactions by the laws of reaction kinetics. By minimizing the error function, we found an appropriate set of parameters under which the numerical simulation of the models shows good agreement with the experimental data [1]. More importantly, the numerical results demonstrate that the inhibition of ATM kinase activity reduces the rate of phosphorylation of DNA-PKcs and hence its release from DSBs, resulting in slower repair, consistent with the experimental observation [1] that complex DSB repair involves DNA-PKcs, but not simple DSB. ATM phosphorylation and autophosphorylation of DNA-PKcs are essential for efficient DSB repair [12] by facilitating release of DNA-PKcs from DNA ends [9].

On the other hand, the proteins involved in NHEJ are generally known, the repair models can be very different, depending on how the detailed repair of different types of DSB is interpreted. Indeed, DSB repair can be modeled in terms of either DNA break filling or through synapsis formation. By break filling, we assume that the DNA DSB ends remain close to each other so that the DSB recruits NHEJ proteins to the ends for rejoining which may involve end filling. This leads to a first order series of reactions and is called a first order model. Break filling is one hypothesis that could result from the observation of the rapid binding of the Ku protein to DNA ends followed by ligation once the ends have been ‘cleaned up’. The situation that DNA free ends do not diffuse significantly apart falls into this scenario. In contrast, we also considered synapsis formation, whereby the DNA free ends recruit NHEJ proteins to form a complex, one at each end and subsequently the two protein complexes meet and form a synapsis [6] for further repair. Modeling synapsis formation involves second order reactions and hence is called a second order model.

Under the “best” set of parameters that we obtain for each type of model by gradient method, we were able to mimic the kinetics of formation and loss of relative fluorescence intensity of the Ku80-EGFP and DNA-PKcs-YFP at DSB on time by numerical simulation of these models. By comparing with the experimental data provided in [1], the numerical simulation of the second order model shows better agreement than that of the first order model with the experimental data in [1], suggesting that the repair of complex DSBs by the NHEJ pathway is more likely a process which occurs through synapsis formation than by break filling. As shown by Goodhead [51] using basic biophysical considerations, the probability of a small volume of diameter less than 10 nm, representative of short segments of DNA, to be intersected by two or more electron tracks produced by X-rays or gamma-rays is negligible below doses of several hundred Gy. Therefore, the ratio of simple to complex DSBs remains constant as dose increases to very large doses [51], and experiments have shown that the yield of DSBs increases linearly with dose up to at least 100 Gy [52]. Our model applies for both physiological conditions of low to moderate doses and for high doses (>10 Gy) often used in experimental investigation and recent models based on DSB repair [27]–[33]. Additionally the rate of repair of DSB induced by 15 or 80 Gy, determined using PFGE, is consistent with the rate of loss of γH2AX after 1 or 2 Gy [13], [53]. Therefore under the experimental conditions used in [1], the repair kinetics of DSBs are constant, consistent with the high copy number of proteins involved in NHEJ [54].

In addition, by switching the synapsis formation and DNA ends processing in the repair of complex DSB, numerical simulations show no significant difference, suggesting that the ordering of synapsis formation and ends processing may not be influential relative to the ligation step. Furthermore, these two processes were therefore merged into one single step, with a new rate denoted by k_EPL_. The resulting model provides a better fit to the data. In this model, both k_EPL_ and k_pD_ are affected by the effect of ATM inhibition, in contrast to only k_pD_ other second order models. When synapsis formation and end processing are merged, not only the ordering between them is unimportant, but also they may occur simultaneously so cannot be kinetically clearly separated. The experimental data showed that an ATM inhibitor reduces the ATM kinase activity by reducing the rate of phosphorylation and as a consequence the rate of release of DNA-PKcs. On the other hand, ATM inhibition also impacts the combined rate k_EPL_ probably because the inhibited ATM activity impairs the rate of phosphorylation of Artemis, which is a substrate of both ATM [49] and DNA-PKcs [6], [42] and usually facilitates DNA end processing in combination with DNA-PKcs. This observation addresses an interesting question from the point of view of modeling of how merging two successive reactions into one affects the short term kinetics and therefore warrants more attention.

Overall, a refined mathematical model has been built based on the dynamics of repair of DSB through NHEJ pathway and supports the pathway dependence on the complexity of the DSB, simple and complex DSBs, leading to the biphasic repair kinetics. We have shown that a SF model is preferred over a simple BF model to explain DSB repair kinetics. Complex DSBs are produced in higher proportion for high linear energy transfer (LET) radiation compared to low LET radiation such as x rays and γ rays [55], [56]. In addition to the higher fraction of complex DSBs, high LET will also produce DSBs in close vicinity to each other whereas low LET radiation is more likely to produce a random distribution of DSBs. The SF model with its inclusion of diffusion of ends prior to synapsis thus provides a straight-forward mechanism to account for the higher effectiveness of high LET radiation in the formation of chromosomal aberrations [55]. In contrast the BF model leaves the increased effectiveness of high LET radiation for chromosomal aberrations for DSB formation in G1 phase of the cell cycle, where NHEJ dominates, largely unexplained. Another consideration is the spectrum of different types of complex DSB involving increased numbers of DNA lesions close to DSB ends with increasing LET. In the current models only a single category of complex DSB has been considered. We expect that the localization of DSB in either euchromatin or heterochromatin may also play role in the biphasic profile, as proposed in [22], [57], and the slower repair of complex DSB may allow alternate pathways to compete for repair [3] especially in the absence of Ku [39]. We will consider these possibilities in future work.

## Supporting Information

File S1 Contains Supplementary Methods on the SF and BF models, Gradient Iteration, and Combination of Two Reactions.
(DOCX)

File S2 Contains description of Data Fitting of DNA-PKcs Foci induced by Fe and C particles, Supplementary Figure S1 and Supplementary Reference S1.
(DOCX)
