# Supplementary material for: Modeling Damage Complexity-Dependent Non-Homologous End-Joining Repair Pathway
Source: PLoS One. 2014 Feb 10;9(2):e85816. doi: 10.1371/journal.pone.0085816 (PMC3919704; doi:10.1371/journal.pone.0085816)
Supplement: File S1 — Contains Supplementary Methods on the SF and BF models, Gradient Iteration, and Combination of Two Reactions. (DOCX) [file pone.0085816.s001.docx]

**Supplementary Material Text S1**

***SF Model - Break Filling***

For a single piece of DNA fragment, one cutoff in the middle results in one break and two pieces of shorter fragments. If the two resulting fragments remain where they are generated without any diffusion, then the break remains and recruits the repair proteins to fill the break for repair. In this paper, we call the model of DNA repair through break filling a BF model. By assuming the concentrations of all the repair proteins remain constants, this BF model leads to a linear system of ODEs whose solution is a linear combination of exponential functions (or exponential function multiplied by a polynomial). From this point of view, the BF model is similar to the double exponential model utilized in [1]. The notations for the first order DSB repair are listed below.

| **Symbol** | $\mathbf{XL}$ | $\mathbf{S}_{\mathbf{0}}$ | $\mathbf{S}_{\mathbf{1}}$ | $\mathbf{S}_{\mathbf{2}}$ | $\mathbf{S}_{\mathbf{3}}$ | $\mathbf{C}_{\mathbf{0}}$ | $\mathbf{C}_{\mathbf{1}}$ | $\mathbf{C}_{\mathbf{2}}$ | $\mathbf{C}_{\mathbf{3}}$ | $\mathbf{C}_{\mathbf{4}}$ | $\mathbf{C}_{\mathbf{5}}$ |
| --- | --- | --- | --- | --- | --- | --- | --- | --- | --- | --- | --- |
| **Complexes** | **XRCC4/Lig IV** | **sDSB** | **sDSB/Ku** | **sDSB/Ku/XL** | **sDSB/XL** | **cDSB** | **cDSB/Ku** | **cDSB/DNAPK** | **End processed** $\mathbf{C}_{\mathbf{2}}$ | $\mathbf{C}_{\mathbf{3}}$**/XL** | $\mathbf{C}_{\boldsymbol{4}}$ **w/o DNAPK** |

The associated reactions are

$S_{0}\underset{\to}{k_{a1} (Ku)} S_{1}$, $S_{1}\underset{\to}{k_{a3} \left( \mathrm{XL} \right)} S_{2}$, $S_{2}\underset{\to}{k_{d1}} S_{3}+Ku$, $S_{3} \underset{\to}{k_{d2}} \mathrm{XL}$,

$C_{0}\underset{\to}{k_{a1}\left( \mathrm{Ku} \right)} C_{1}$, $C_{1} \underset{\to}{k_{a2}(\mathrm{DNA}-\mathrm{PKcs})} C_{2} C_{2} \underset{\to}{k_{\mathrm{EP}}} C_{3}$, $C_{3} \underset{\to}{k_{a3}(XL)} C_{4}$, $C_{4} \underset{\to}{k_{\mathrm{pD}}}C_{5}+DNAPK, C_{5} \underset{\to}{k_{d2}}\mathrm{XL}$.

where $k_{a1}$, $k_{a2}$and $k_{a3}$ are the recruitment rates of Ku, DNA-PKcs and XL to the DSB, respectively, $k_{d1}$ and $k_{d2}$ the release rates of Ku and XL from DSB, $k_{\mathrm{EP}}$the end processing rate and $k_{\mathrm{pD}}$ the phosphorylation rate of DNA-PKcs.

The stoichiometric matrices and reaction fluxes are

$M_{S}=\left[ \begin{matrix} \begin{matrix} -1 & 0 \\ 1 & -1 \end{matrix} & \begin{matrix} 0 & 0 \\ 0 & 0 \end{matrix} \\ \begin{matrix} 0 & 1 \\ 0 & 0 \end{matrix} & \begin{matrix} -1 & 0 \\ 1 & -1 \end{matrix} \end{matrix} \right], R_{S}=\left[ \begin{matrix} \begin{matrix} k_{a1}S_{0} \\ k_{a3}S_{1} \end{matrix} \\ \begin{matrix} k_{d1}S_{2} \\ k_{d2}S_{3} \end{matrix} \end{matrix} \right]$, $M_{C}=\left[ \begin{matrix} \begin{matrix} -1 & 0 & 0 \\ 1 & -1 & 0 \\ 0 & 1 & -1 \end{matrix} & \begin{matrix} 0 & 0 & 0 \\ 0 & 0 & 0 \\ 0 & 0 & 0 \end{matrix} \\ \begin{matrix} 0 & 0 & 1 \\ 0 & 0 & 0 \\ 0 & 0 & 0 \end{matrix} & \begin{matrix} -1 & 0 & 0 \\ 1 & -1 & 0 \\ 0 & 1 & -1 \end{matrix} \end{matrix} \right], R_{C}=\left[ \begin{matrix} \begin{matrix} k_{a1}C_{0} \\ k_{a2}C_{1} \\ k_{\mathrm{EP}}C_{2} \end{matrix} \\ \begin{matrix} k_{a3}C_{3} \\ k_{\mathrm{pD}}C_{4} \\ k_{d2}C_{5} \end{matrix} \end{matrix} \right]$,

Then the amount of foci of Ku and DNA-PKcs are

$$S_{K}=S_{1}+S_{2}+C_{1}+C_{2}+C_{3}+C_{4}=S_{1}+S_{2}+C_{1}+C_{D}, C_{D}=C_{2}+C_{3}+C_{4}$$

and

$$\frac{dS_{K}}{\mathrm{dt}}=k_{a1}S_{0}-k_{d1}S_{2}+k_{a1}C_{0}-k_{\mathrm{pD}}C_{4}, \frac{dC_{D}}{\mathrm{dt}}=k_{a2}C_{1}-k_{\mathrm{pD}}C_{4}.$$

Apparently the dynamics of both $S_{K}$ and $C_{D}$ foci are independent of the release rate $k_{d2}$of XL, which, however, accounts for the DSB repair kinetics. Because the BF model is a linear system, its solution is the linear combination of exponential function or exponential function multiplied by a polynomial, including double exponential model as used in [1].

***SF Model - Synapsis Formation***

The stoichiometric matrices and reaction fluxes of the synapsis formation (SF) model are given by

$R_{S}=\left[ \begin{matrix} \begin{matrix} k_{a1}S_{0} \\ {\frac{1}{2}k}_{\mathrm{LS}}S_{1}^{2} \end{matrix} \\ \begin{matrix} k_{d1}S_{2} \\ k_{d2}S_{3} \end{matrix} \end{matrix} \right]$, $R_{C}=\left[ \begin{matrix} \begin{matrix} k_{a1}C_{0} \\ k_{\mathrm{EP}}C_{1} \\ {\frac{1}{2}k}_{\mathrm{LD}}C_{2}^{2} \end{matrix} \\ \begin{matrix} k_{a1}C_{3} \\ k_{\mathrm{pD}}C_{4} \\ k_{d2}C_{5} \end{matrix} \end{matrix} \right]$

where

$$M_{S}=\left[ \begin{matrix} \begin{matrix} -1 & 0 \\ 1 & -2 \end{matrix} & \begin{matrix} 0 & 0 \\ 0 & 0 \end{matrix} \\ \begin{matrix} 0 & 1 \\ 0 & 0 \end{matrix} & \begin{matrix} -1 & 0 \\ 1 & -1 \end{matrix} \end{matrix} \right], M_{C}=\left[ \begin{matrix} \begin{matrix} -1 & 0 & 0 \\ 1 & -1 & 0 \\ 0 & 1 & -2 \end{matrix} & \begin{matrix} 0 & 0 & 0 \\ 0 & 0 & 0 \\ 0 & 0 & 0 \end{matrix} \\ \begin{matrix} 0 & 0 & 1 \\ 0 & 0 & 0 \\ 0 & 0 & 0 \end{matrix} & \begin{matrix} -1 & 0 & 0 \\ 1 & -1 & 0 \\ 0 & 1 & -1 \end{matrix} \end{matrix} \right].$$

If the NHEJ proteins are assumed to be constant, then the repair of simple and complex DSBs can be decoupled as

${\dot{\mathbb{X}}}_{S}={M_{S}R_{S}(\mathbb{X}}_{S}), {\dot{\mathbb{X}}}_{C}={M_{C}R_{S}(\mathbb{X}}_{C})$.

Because matrix $\mathbb{SM}$ is a fully ranked square matrix, we have $\mathbb{SM}v=0$ implies $v=0$. This also applies to the BF model.

For both BF and SF models, when the production kinetics of DSB, as the radiation being applied, is taken into account, we have two more reactions

$$D_{R} \underset{\to}{b_{K}} S_{0}, D_{R} \underset{\to}{b_{C}} C_{0}$$

where $D_{R}$ is the dose rate, $b_{S}$ and $b_{C}$ are the production rates of simple and complex DSB by radiation. We may either use augmented stoichiometric matrix and reaction fluxes to incorporate these two reactions into the system

$\bar{M}_{S}=\left[ \begin{matrix} v_{S} & M_{S} \end{matrix} \right]$, $\bar{R}_{S}=\left[ \begin{matrix} b_{S}D_{R} \\ R_{S} \end{matrix} \right]$, $\bar{M}_{C}=\left[ \begin{matrix} v_{C} & M_{C} \end{matrix} \right]$, $\bar{R}_{C}=\left[ \begin{matrix} b_{C}D_{R} \\ R_{C} \end{matrix} \right]$,

or treat them as external controls,

$$u_{S}=\left[ \begin{matrix} b_{S}D_{R} \\ 0_{4\times1} \end{matrix} \right], u_{C}=\left[ \begin{matrix} b_{C}D_{R} \\ 0_{5\times1} \end{matrix} \right],$$

which is more convenient for dynamical analysis. Here, by introducing these controls into the system, we have the complete model

${\dot{\mathbb{X}}}_{S}={\bar{M}_{S}\bar{R}_{S}(\mathbb{X}}_{S})={M_{S}R_{S}(\mathbb{X}}_{S})+u_{S}\left( t \right), {\dot{\mathbb{X}}}_{C}={\bar{M}_{C}\bar{R}_{C}(\mathbb{X}}_{C})={M_{C}R_{S}(\mathbb{X}}_{C})+u_{C}(t)$. (S1)

Or the entire system can be collectively written as

| $\dot{\mathbb{X}}=\frac{d\mathbb{X}}{\mathrm{dt}}=\mathbb{SM} \mathbb{R}(\mathbb{X}, p, t)$ | (S2) |
| --- | --- |

where stoichiometric matrix and reaction flux vector for the model are given by

$$\mathbb{SM}=\left[ \begin{matrix} \bar{M}_{S} & 0 \\ 0 & \bar{M}_{C} \end{matrix} \right], \mathbb{R}=\left[ \begin{matrix} \bar{R}_{S} \\ \bar{R}_{C} \end{matrix} \right].$$

***Gradient Iteration***

Consider the reaction rate equation (S2). Then the amount of foci Ku80-EGFP and DNA-PKcs-YFP can be defined as

$F_{K}=\left\langle\mathbb{X},v_{K} \right\rangle, F_{D}=\left\langle\mathbb{X},v_{D} \right\rangle$,

respectively, where $\left\langle, \right\rangle$ is inner product of two vectors, $v_{K}$ and $v_{D}$ are associated vectors pointing to the species containing the respective foci. For example, $v_{K}=(0,1,2,0,0,1,1,2,2,0)$ and $v_{D}=\left( 0,0,0,0,0,1,1,2,2,0 \right)$ for the SF Model 3.

To find a proper parameter set $p$ so that the associated numerical solution of (S2) fits the data sets $\left\{ (t_{K}^{i}, K_{i}) \right\}_{i=1}^{N_{K}}$ and $\left\{ (t_{D}^{j}, D_{j}) \right\}_{j=1}^{N_{D}}$, we apply gradient method. Starting with an initial estimate $p^{0}$, the gradient iteration is given by

$p^{i+1}=p^{i}+h\nabla E(p^{i})$,

where $E$ is the error function measuring the difference between the numerical solution of (S2) and the given data sets, and it is defined by

$E\left( p \right)=E\left( q, k_{\mathrm{pD}} \right)+\tilde{E}\left( q, \tilde{k}_{\mathrm{pD}} \right)=E_{K}\left( q,k_{\mathrm{pD}} \right)+E_{D}\left( q, k_{\mathrm{pD}} \right)+\tilde{E}_{K}\left( q, \tilde{k}_{\mathrm{pD}} \right)+\tilde{E}_{D}\left( q, \tilde{k}_{\mathrm{pD}} \right)$,

$\nabla E(p)$ is the gradient of error function $E$ at point $p$, $h$ is the iteration step, also called learning rate. As defined in the main text, each term on the right side of error function takes the same form of $L^{2}$ norm, for simplicity, we will consider only $E_{K}\left( q,k_{\mathrm{pD}} \right)$ as an example. Similar calculation applies to other terms. First we have

$\frac{\partial E_{K}}{\partial p}=\left( \frac{1}{E_{K}} \right)\sum_{i=1}^{N_{K}} \left[ K\left( t_{K}^{i}, p \right)-K_{i} \right](t_{K}^{i}-t_{K}^{i-1})\left( \frac{\partial}{\partial p}K\left( t_{K}^{i}, p \right) \right)$.

Assume that for fixed parameter set $p$, function $F_{K}\left( t \right)$ reaches its maximum at time $t_{K}(p)$, then

$\frac{\partial}{\partial t}F_{K}\left( t_{K}\left( p \right),p \right)=0$.

Set $F_{\mathrm{KM}}(p)=F_{K}\left( t_{K}\left( p \right),p \right)$, then by definition, $K\left( t \right)=\frac{F_{K}\left( t,p \right)}{F_{\mathrm{KM}}(p)}$ and

$\frac{dF_{\mathrm{KM}}}{dp}=\left[ \frac{\partial}{\partial t}F_{K}\left( t_{K}\left( p \right),p \right) \right]\left( \frac{\partial t_{K}}{\partial p} \right)+\left[ \frac{\partial}{\partial p}F_{K}\left( t_{K}\left( p \right),p \right) \right]=\frac{\partial}{\partial p}F_{K}\left( t_{K}\left( p \right),p \right)$.

Therefore, we have

$\frac{\partial K}{\partial p}=\frac{\left[ \frac{\partial}{\partial p}F_{K}\left( t,p \right) \right]F_{\mathrm{KM}}(p)-F_{K}\left( t,p \right)\left[ \frac{\partial}{\partial p}F_{K}\left( t_{K}\left( p \right),p \right) \right]}{{[F_{\mathrm{KM}}(p)]}^{2}}$,

in which, $\frac{\partial}{\partial p}F_{K}\left( t,p \right)$ can be obtained by solving the following equation

| $\frac{d}{\mathrm{dt}}\left( \frac{\partial\mathbb{X}}{\partial p} \right)=\mathbb{SM} \left[ \left( \frac{\partial\mathbb{R}}{\partial\mathbb{X}} \right)\left( \frac{\partial\mathbb{X}}{\partial p} \right)+\left( \frac{\partial\mathbb{R}}{\partial p} \right) \right]$ | (S3) |
| --- | --- |

where $\left( \frac{\partial\mathbb{R}}{\partial\mathbb{X}} \right)$ is the Jacobian matrix.

***Combination of Two Reactions***

Consider the following reaction system

$x\underset{\Rightarrow}{a} y$, $y\underset{\Rightarrow}{b}z$, (S4)

where the arrow “⇒” reflects only the transition from the reactant to the product without specifying the stoichiometry. Then by the law of chemical reactions (mass action, Michaelis-Menten, etc.), the reaction rate equation for this reaction system is given by

$\frac{dx}{dt}={-aR}_{1}\left( x \right), \frac{dy}{dt}=aR_{1}\left( x \right)-{bR}_{2}\left( y \right), \frac{dz}{dt}={bR}_{2}\left( y \right),$ (S5)

where $a>0$ and $b>0$ are reaction rate constants, ${aR}_{1}$and $bR_{2}$ are nonnegative continuous functions representing reaction fluxes of the two reactions and satisfy

$R_{1}\left( 0 \right)=0, R_{2}\left( 0 \right)=0$.

Note that the reaction system has a conservation law

$x+y+z=x\left( 0 \right)+y\left( 0 \right)+z\left( 0 \right)=x(0)$,

if we assume the initial concentrations of intermediate species $y$ and the product $z$ are zeros. Additionally by the positiveness of the solution, we know that the solution of system (S5) is bounded for all $t\geq0$. For any fixed $a>0$, by the rescaling of time $\tau=bt$, the reaction rate equation of species $y$ can be rewritten as

$\frac{dy}{d\tau}=\frac{a}{b}R_{1}\left( x \right)-R_{2}\left( y \right).$ (S6)

The boundedness of both $y(\tau)$ and $\frac{dy}{d\tau}$ ensures the uniform convergence of $y\left( \tau\right)$ to a limit, say $y_{\infty}(t)$, as $b\to\infty$, and $y_{\infty}(t)$ satisfies $\frac{dy}{d\tau}=-R_{2}\left( y \right).$ Consequently $y_{\infty}\left( \tau\right)=0$ by initial condition $y\left( 0 \right)=0$. Moreover, note that

$\frac{d(y+z)}{dt}=aR_{1}\left( x \right)\Rightarrow\frac{d(y_{\infty}+z_{\infty})}{dt}=aR_{1}\left( x_{\infty} \right)$.

Then it follows that as $b\to\infty$, system (S1) approaches

$\frac{dx}{dt}={-aR}_{1}\left( x \right), \frac{dz}{dt}={bR}_{2}\left( y \right),$ (S7)

which is the reaction rate equations for the reaction system

$x\underset{\Rightarrow}{a} z$. (S8)

In other words, if the reaction rate $b$ of the second reaction is large enough, reaction system (S4) can be approximated by a single reaction (S8).
