# Supplementary material for: Modeling Damage Complexity-Dependent Non-Homologous End-Joining Repair Pathway
Source: PLoS One. 2014 Feb 10;9(2):e85816. doi: 10.1371/journal.pone.0085816 (PMC3919704; doi:10.1371/journal.pone.0085816)
Supplement: File S2 — Contains description of Data Fitting of DNA-PKcs Foci induced by Fe and C particles, Supplementary Figure S1 and Supplementary Reference S1. (DOCX) [file pone.0085816.s002.docx]

**Supplementary Material S2**

***Data Fitting of DNA-PKcs Foci induced by Fe and C Particles***

In this section, we compared the model with data of DNA-PKcs foci induced by Fe and C particles that were provided in [S[1](#_ENREF_1)]. The data showed the kinetics of DNA-PKcs autophosphorylation in plateau-phase HFL III cells irradiated with Fe (~200keV/μm) and C (~70keV/μm) particles separately. For more details about the experiments, please see [S[1](#_ENREF_1)].

**
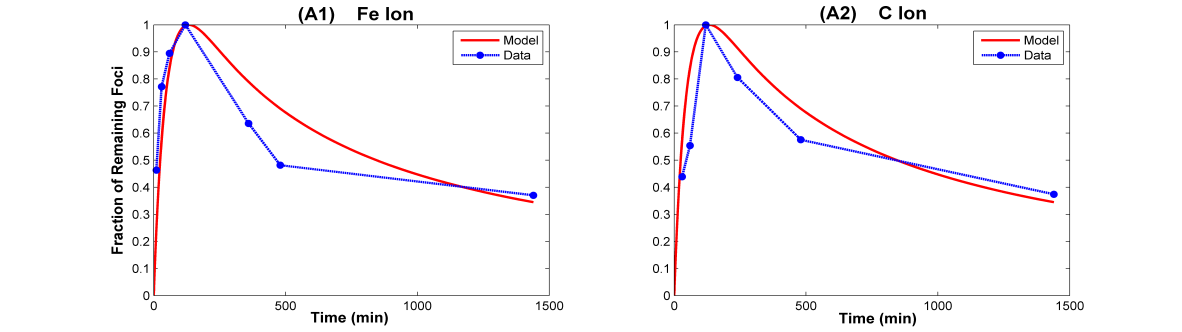
**

Figure S1. Comparison of SF Model with Fe and C Ion-induced DNA-PKcs Foci.

Because DNA-PKcs participates only in the complex DSB repair, its kinetics is determined by only four parameters

($b_{C},k_{a1}, {k_{\mathrm{EPL}}, k}_{\mathrm{pD}}$). To fit the data as shown in Figure S2, we have

$$b_{C}^{\mathrm{Fe}}=1.4800, k_{a}^{\mathrm{Fe}}=0.035, k_{\mathrm{EPL}}^{\mathrm{Fe}}=0.0026, k_{\mathrm{pD}}^{\mathrm{Fe}}=0.0219$$

and

$$b_{C}^{C}=0.7820, k_{a}^{C}=0.0251, k_{\mathrm{EPL}}^{C}=0.0042, k_{\mathrm{pD}}^{C}=0.0375.$$

The above comparison with different experimental data set further validates our model by capturing the kinetic profile of DNA-PKcs foci. Moreover, the numerical analysis shows that Fe ion has higher production rate of complex DSB than C ion, which, in turn, has higher rate than laser beam.

**Supplementary Reference**

S1. Okayasu, R., et al., (2006) Repair of DNA damage induced by accelerated heavy ions in mammalian cells proficient and deficient in the non-homologous end-joining pathway*.* Radiat Res. 165(1): 59-67.
